# Cohort and gender differences in the association between childlessness and social exclusion in old age

**DOI:** 10.1007/s10433-024-00831-z

**Published:** 2024-11-18

**Authors:** Julia Sauter, Iuliana Precupetu, Marja Aartsen

**Affiliations:** 1 Institute of Social Sciences - LIVES, University of Lausanne, Bâtiment Géopolis, CH-1015 Lausanne, Switzerland; 2 Research Institute for Quality of Life, Romanian Academy, Bucharest, Romania; 3 Research Institute of the University of Bucharest, University of Bucharest, Bucharest, Romania; 4 NOVA - Norwegian Social Research, Oslo Metropolitan University, Oslo, Norway

**Keywords:** Social exclusion, Quantitative, Childlessness, Ageing

## Abstract

Social exclusion (SE) and its detrimental health outcomes are a key concern in European policies. This study investigates whether childless older adults face a higher risk of SE compared to those with children, how these potential differences have evolved, and whether SE among childless older men differs from that experienced by childless older women. Children are perceived in most cultures as an insurance of social integration in old age. Delayed childbearing and falling birth rates in Western countries have raised concerns about the old age of those who remain childless and reach the last decades of their lives more socially excluded. The exclusionary risks of not having children may be gendered and different across time, but research on this topic is underdeveloped. The analysis utilizes data from the first (2002) and ninth (2018) rounds of the European Social Survey (ESS), focusing on participants aged between 65 and 74. The study reveals that childless older adults have fewer social meetings and engage in fewer social activities than parents. Findings are robust concerning time and gender, as the disadvantage of not having children compared to those with children is similar over time and for men and women. The results highlight that childless older adults face an elevated risk of SE.

Social exclusion (SE), or the inability to participate in the normal relationships and activities available to most people, is a key concern of European social policy. It affects both the quality of life of individuals and the equity and cohesion of society as a whole (Levitas et al. 2007). Despite recommendations of the European Commission to reduce the number of socially excluded people (European Commission 2008), almost one-fifth of the 65 + people in Europe were still at increased risk of economic and social exclusion in 2021, with higher percentages of exclusion for women than men (Statistics Eurostat, online data code ILC_PEPS01N). While older people have slightly lower risks than younger age groups to be excluded, they tend to be excluded longer (Scharf and Keating 2012), suggesting a more considerable impact of SE in later life.

Social exclusion has long been defined as a condition that mainly concerns the economic conditions (poverty, material deprivation, or living in a household with very low work intensity, European Commission 2010), but scholars increasingly converge in their opinion that social exclusion includes various dimensions, among which social relations (Levitas et al. 2007; Walsh et al. 2017; Dahlberg et al. 2022). However, empirical support for the social dimension, which is the focus of this study, is still limited. Research on SE identified individual characteristics such as age, gender, race, ethnicity, socio-economic status, sexual orientation, and health, and to a limited extent, contextual factors such as national regulations, ageism, or neighbourhood characteristics to be related to SE (Scharf et al. 2021). Adult children may protect older people from SE as they have been perceived in most cultures as insurance of social integration in old age and parenthood as a “key organizer” of the life course (Dykstra and Hagestad 2007). However, trends in delayed childbearing and falling birth rates in Western countries have raised concerns about the old age of those who do not become parents and, therefore, reach the last decades of their lives with potential deficits in the number of social relations.

Social exclusion can be characterized by four common features (Walsh et al. 2017). It is relative, as it describes the situation of a person compared to the mainstream society; dynamic, as the degree to which people are excluded or included can vary over the life course; it involves agency, which includes both the situation in which people are excluded involuntarily and situations in which older people choose to exclude themselves, and SE is multidimensional as people can be excluded from one or more domains at the same time. Domains that are typically distinguished include neighbourhood and community, social and health services, material and financial resources, social relations, and civic participation (Levitas et al. 2007; Walsh et al. 2017).

This study focuses on two of the social aspects of SE, i.e. social meetings and civic participation. More precisely, it will address the role of childlessness in SE in later life and how these associations might be gendered and vary over time. It questions whether childless older individuals are more prone to SE than those with children. Furthermore, based on the destandardization of the life course of later-born cohorts and potential changes in gender-related task differentiation, it questions whether differences between childless people and parents vary by gender and historical time. We investigate whether childless people from an earlier-born cohort were more socially excluded in later life than people from a later-born cohort and whether SE for childless men differs from childless women.

## Theoretical background

### Childlessness and social exclusion in later life

A suitable perspective for understanding the role of children in later-life SE and how that might have changed for later-born cohorts is the life course theory (Elder 1998). A key principle of the life course theory is that of “linked lives”, acknowledging that human lives are embedded in social relations such as kin and friends and that social regulation occurs in part through these relationships. Another fundamental principle in life course theory is that of “time and place”. Social processes are partly determined by the historical context and normative expectations in which they occur. Historical shifts in the number of children may have impacted normative expectations and opportunities for social participation. Although there is little consideration of the role of gender in Elder’s life course theory, other researchers who have also adopted the life course perspective highlighted important gender differences in life course developments (e.g. Hagestad and Dykstra 2016).

An extensive body of research has documented the beneficial effects of high levels of social interaction between adult children and their older parents (Thomas et al. 2017; Connidis and Barnett 2018; Rossi and Rossi 2018). Not having children is often considered a lack of crucial relationships necessary for social integration in old age. Although childless older adults can develop strategies to overcome this lack, such as relying more on extended kin or friends (Schnettler and Wöhler 2016; Deindl and Brandt 2017), many studies have shown that childless older adults have smaller networks than those with children (Dykstra and Wagner 2007; Schnettler and Wöhler 2016), fewer opportunities to socially connect and less close relationships (Albertini and Kohli 2009), leading to enhanced loneliness in later life (Zoutewelle-Terovan and Liefbroer 2018).

Childless older adults form a heterogeneous group of people, including those who choose to be childless and those who cannot have children for biological or other reasons. Despite this heterogeneity, the consequences for SE may be similar for structural, normative, or functional reasons. Firstly, a life course without children is often dominated by working careers and contacts with same-aged friends and siblings (Connidis and McMullin 1992, 1999). While these contacts are valuable for social integration, they also make older people more vulnerable to social isolation at higher ages, as once connections with friends are lost, they are not likely to be replaced by new ones. Hence, not having children reduces both vertical familial structure and the wider formal and informal social links children can bring (Hadley 2018), irrespective of their pathway to childlessness.

The distinction between voluntary and involuntary childlessness is often blurred, reflecting a complex interplay of personal choice, societal expectations, and circumstantial factors. Voluntary childlessness typically refers to individuals or couples who consciously decide not to have children, often due to personal preferences, career aspirations, or lifestyle choices (Kelly 2009). Involuntary childlessness, on the other hand, usually involves those who desire children, but are unable to have them due to biological, medical, or circumstantial reasons, such as infertility or lack of a suitable partner. However, the line between these categories can be indistinct. For instance, some may initially choose childlessness voluntarily, but later experience regret or a change in circumstances that make parenthood more appealing or feasible (Letherby 2002). Additionally, societal pressures and stigmatization can influence how individuals perceive and categorize their own childlessness, sometimes leading to a reinterpretation of their situation as either voluntary or involuntary (Park 2005). Not being in the normative category either by choice or involuntarily can reduce social opportunities, with negative consequences for the number of social relations and social activities. Finally, not having children means not having access to a primary resource that supports social integration once people become dependent on others to maintain social relations and participate in society. We, therefore, expect that older childless individuals are more socially excluded than older parents (H1).

### Cohort differences in the association between childlessness and social exclusion

As a result of the progressive institutionalization of Western societies during the first half of the twentieth century, socially standardized life trajectories were established, in which specific transitions were expected to be made by a large majority of society. In this process, age became central to organizing the course of life into a defined sequence of ordered stages. Individuals arrange their lives according to a social clock that dictates the age at which it is appropriate to make each socially valued transition in the social and historical context in which they fit and thus endorse different roles (Riley et al. 1972; Settersten 2003), for example becoming active in the labour market, becoming a spouse, a parent, or a retiree.

Normative transitions experienced by most individuals at different ages have a temporality, implying potential social sanctions for those who carry them out too early, too late, or not at all (Hareven and Masaoka 1988). Informal mechanisms of social control reinforce age-related prescriptions and lead to their internalization by individuals (Settersten 2003). In this context, the major transitions that punctuate the life course are expected events by society and individuals (Hareven and Masaoka 1988; Kohli 2007). Therefore, much of the literature on normative life course transitions has focused on the perceived adverse consequences of deviation from the prescribed social timeline. Many studies (see Mayer 2009 for a summary) have assumed that a non-normative trajectory would have adverse long-term effects on individuals’ integration into society (Furstenberg 2005). During the time when cohorts born in the early decades of the twentieth century reached adulthood, the transition to parenthood held immense social and institutional significance (Sánchez-Barricarte 2018). Becoming a parent was highly esteemed within the societal framework of that era. It was conventionally linked to marriage, and as a result, most individuals followed this traditional pathway. Notably, childlessness rates were at an unprecedented low during this period, emphasizing the prevailing norm of parenthood in conjunction with marital union (Duvoisin et al. 2016).

In later-born cohorts, the normative pressure to transition to parenthood diminished as life courses and family trajectories deviated from standardized patterns in the late 1960s (Widmer and Ritschard 2009). However, it may not have offset the disadvantages of being childless in earlier-born cohorts, as recent work on childlessness found that childless older people still have fewer opportunities to socially connect (e.g. Kjær and Siren 2021; Fihel et al. 2022). This shift towards increased complexity and diversity in life paths has been highlighted by individualization theorists, who view it as a profound societal transformation affecting a significant portion of individuals' personal lives throughout the twentieth century (Beck and Beck-Gernsheim 1994; Sennett 1998; Beck 2016). Based on these observations, we anticipate that childless individuals from earlier-born cohorts may experience more SE than those from later-born cohorts (Hypothesis 2). The greater normative acceptance and recognition of diverse life trajectories in later cohorts potentially reduce the social stigma and exclusion experienced by those who did not become parents.

### Gender differences in the association between childlessness and social exclusion

Gender differences in the link between childlessness and SE are frequently attributed to variations in the private and public responsibilities assumed by men and women (Levy et al. 2002, 2013). Gender roles often lead to divergent responsibilities for women and men regarding family and work domains. Women with children often shoulder private tasks, limiting their economic and social participation opportunities. On the other hand, men tend to focus on public tasks and careers. However, these gender differences may not necessarily hold for individuals without children. For women, not having children has been described as an "ultimate liberation" from caregiving responsibilities, enabling them to engage in societal activities fully (Peterson 2015). Nevertheless, the reality is more complex, as societal norms idealizing motherhood can also subject childless women to a higher risk of stigmatization and SE (Letherby 2002; Turnbull et al. 2016).

Conversely, for men, the influence of family transitions (such as marital transitions and the transition to parenthood) on well-being is not as pronounced as the impact of employment and professional careers (Levy et al. 2002, 2013). Additionally, the effect of childlessness among men is conditioned to a much larger extent by partner status than by not having children. For example, Dykstra (2006) found that men and women without children had smaller social networks in a sample of older Dutch adults. Still, for men, differences became insignificant when controlled for partner status, which was not the case for women. In terms of health, Kendig et al. (2007) showed that never-married and formerly married childless men were more likely than married, childless men to report being in poor physical health, whereas, among childless women, there were no significant differences in self-reported health based on partner status. Other studies have shown that the quality of life outcomes of never-married childless women are more favourable than those of their married counterparts (Koropeckyj-Cox and Call 2007; Keizer and Ivanova 2017; Keizer et al. 2008).

Taken together, these findings suggest that the presence of a partner can compensate for the impact of childlessness on SE for men, but not for women. We, therefore, hypothesize that childless women are more socially excluded than childless men (H3). With regard to partner status, we hypothesize that overall, those childless older adults with a partner are less socially excluded than those without a partner (H4).

### Gender-specific cohort differences in the association between childlessness and social exclusion

In the previous section on cohort differences in the association between childlessness and SE, we argued that childless individuals from earlier-born cohorts might experience more SE as compared to parents than those from later-born cohorts, but we did not anticipate any gender differences. However, there may be good reasons to expect that potential cohort differences in SE and having children or not are different for men and women. For example, over the last decades, the gendered division of labour and household work has become less pronounced due to secularization and improved childcare provisions (Firat et al. 2023; Bambra 2004). Consequently, paid working time decreased for male and increased for female workers, while time dedicated to household work and thus opportunities to connect with other people through the children fell for women and slightly rose for men (Kan et al. 2011). In other words, if this gender convergence holds, the hypothesized disadvantage for childless people compared to parents will diminish for women (H5a) and increase for men (H5b).

## Methods

### Data

Data come from the European Social Survey (ESS), a cross-national survey of attitudes and behaviour conducted across Europe biennially since 2001 (https://www.europeansocialsurvey.org/). ESS is a pan-European cross-sectional study covering a total time span of 18 years. Data are collected via face-to-face CAPI interviews. The repeated cross-sectional design and the extensive time span make the ESS particularly well suited for investigating differences between same-aged cohorts born at different points in time. With each new wave, new probability samples are drawn representing all persons aged 15 and over residing in private households.

The analysis included all available countries in the ESS. However, to maintain consistency and comparability in our analysis, we excluded post-communist countries and those without a distinct baby boom period (e.g. Portugal) (Van Bavel and Reher 2013). Contrary to Western societies, fertility transition pathways of post-communist countries are characterized by stable fertility rates in the studied birth cohorts (namely between the 1920s and 1960s) (Frejka 2017). We did not exclude Eastern Germany, as it experienced similar fertility patterns to Western Germany during and after the Second World War (Dorbritz 2008). Additionally, we excluded countries that did not participate in both survey rounds (e.g. Luxembourg, Israel, Cyprus, Iceland) to ensure robust comparisons. The final set of countries included in our study were Austria, Belgium, Switzerland, Germany, Denmark, Spain, Finland, France, the UK, Ireland, Italy, the Netherlands, Norway, and Sweden.

Following the European Social Survey (ESS, 2014) recommendations, we employed post-stratification weights in combination with population size weights (anweight) to account for sample representativeness. For Cohort 1, the weighted sample size was 3,188; for Cohort 2, it was 4,115. In both cohorts, the number of female participants was slightly higher than that of male participants, with 53.1% female participants in Cohort 1 and 52.5% in Cohort 2 (see Table 1).

**Table 1:** Descriptive statistics of the study sample, by cohort, gender, and childlessness (weighted data)

	Had children	Childless	Sig. Of Δ	Cohort 1, born between 1929 and 1937	Cohort 2, born between 1945 and 1953	Sig. Of Δ	Men	Women	Sig. Of Δ
Ever had children living in the household (% yes)				78.9	79.1		77.6	80.2	**
Frequency of socially meeting with friends, relatives or colleagues (Mean, 1–7)	4.85	4.68	**	4.71	4.89	**	4.82	4.80	
Take part in social activities compared to others of same age (Mean, 1–7)	2.76	2.65	**	2.59	2.84	**	2.75	2.72	
Age of respondent (Mean, years)	69.29	69.44	*	69.38	69.26	*	69.38	69.26	*
Gender (% female)	53.6	49.7	**	53.1	52.5				
Highest level of education ISCED (Mean, 1–5)	2.55	2.47	**	2.14	2.84	**	2.75	2.34	**
Has a partner (% yes)	72.7	54.3	**	67.0	70.1	*	76.5	62.0	**
Subjective health (Mean, 1–5)	3.46	3.44		3.39	3.51	**	3.51	3.41	**
Hampered in daily activities (Mean, 1–3)	2.55	2.56		2.53	2.56		2.56	2.54	
Migrant background (% yes)	6.9	7.6		5.6	8.1	**	6.9	7.1	
N	5770	1534		3188	4115		3449	3854	

Sig. Of Δ = Significance of difference (based on T-test for Means, Chi-squared test for percentages), * = *p* < 0.05, **p < 0.01. Countries included Austria, Belgium, Switzerland, Germany, Denmark, Spain, Finland, France, UK, Ireland, Italy, the Netherlands, Norway, and Sweden

### Dependent variables

The assessment of SE was based on two questions: “How often do you socially meet with friends, relatives or colleagues” with answering categories ranging from 1 (never) to 7 (every day) and “How often do you take part in social activities compared to others of same age”, with answering categories ranging from 1 (much less than most) to 5 (much more than most). While both the two dependent variables can indicate SE, we did not use an index because of the concepts’ heterogeneity. The first concerns informal social networks in the community (friends, colleagues, and relatives), whereas the second refers to associational participation. Involvement in these spheres may depend on different factors (Gallie et al. 2003), which we would be unable to detect whether we had used an index. To compare the effect sizes between both outcomes, we standardized both variables for the analysis.

### Independent variables

The key independent variable of interest is childlessness. For “ever had at least one child”, we combined “Are there children living in the household” and “Have you ever had any children of your own, stepchildren, adopted children, foster children or a partner’s children living in your household?”. Answers were combined into a dichotomized score with categories “yes, ever had or still have a child” (1) and “no, never had a child” (0). For Round 9, information about children currently in the household was derived from information on all types of relations in the household, and if at least one household member was a child (including own child, stepchildren, adopted children, foster children, and children of the partner), it was counted as having children in the household.

## Control variables

We included education, health, being hampered in daily activities (HDA), and migrant status in our analytical models to control for potential spurious relations. Lower education is related to higher levels of SE, maybe through the opportunities a higher education provides to avoid poverty and maintain social relationships (Aartsen et al. 2021). At the same time, the level of education increased over time, particularly for women (Jalovaara et al. 2019). We also included health and HDA since bad health and limitations in daily activities may enhance SE. Prior research showed that migrant background is associated with differential levels of SE compared to natives (Lui Gallassi and Harrysson 2021); that is why we included migrant status as a control variable.

The level of education reflects the International Standard Classification of Education, and the number of categories was reduced and recoded such that it consists of 5 levels; Less than lower secondary education (ISCED 0–1), lower secondary education completed (ISCED 2), upper secondary education completed (ISCED 3), post-secondary non-tertiary education completed (ISCED 4), and tertiary education completed (ISCED 5–6). Subjective general health is a five-point scale ranging from 1 to 5, which we recoded such that higher scores indicate better subjective health. HDA was evaluated with a single question, “Are you hampered in your daily activities in any way by any longstanding illness, or disability, infirmity or mental health problems?” with three answering categories: (1) yes, a lot, (2) yes, to some extent, and (3) no. Migration background is based on whether people were born in the country where they live at the time of the interview, which was recoded into no (1) and yes (0). Finally, we categorized countries into four groups, “conservative” (including Austria, Belgium, Switzerland, Germany, France, and the Netherlands), “social-democratic” (including Denmark, Finland, Norway, and Sweden), “liberal” (including Ireland and the UK), and “Southern European” (including Spain and Italy) (Firat et al. 2023).

Finally, we included the type of welfare state in our models to control for the potential gender differences in norms towards parenthood and the speed or timing of a change in these norms. For example, Norway, Sweden, Denmark, and Finland (social-democratic states) were all pioneers in promoting gender equality and providing extensive support for families, influencing norms towards having fewer children. In contrast, countries with strong familistic traditions, such as Italy and Spain, all experienced a slower shift in norms, but eventually also saw declining birth rates and changing family norms in the post-war period. While it can be argued that differences in norms and changes in norms can differ between countries, we expect that variations within one welfare regime are small. Moreover, as we have only 15 countries a multi-level analysis, we may not have sufficient statistical power to detect robust and reliable differences. Rather than considering multi-level models, and for reasons of parsimony, we added the four welfare state clusters to the models to control for a potential impact of the welfare state.

With regard to partnership status, for Round 1, we dichotomized marital status into having a partner (1) and no partner (0), which includes the categories “separated”, “divorced”, “widowed”, and “never married”. “Refusal”, “don’t know”, and “no answer” are treated as missing values. For Round 9, we had to harmonize the information about marital status with Round 1, and in line with recommendations of ESS, we combined marital status (*marsts*) with the relationship with current husband, wife, or partner (*rshpsts*). Gender is coded 1 (male) and 2 (female).

### Analytical approach

We are interested in analysing two mechanisms: 1) norms (in our case, the transition to parenthood) can stigmatize individuals who do not conform, leading to SE, and 2) this stigmatization can have long-lasting effects. To empirically test these mechanisms, we conduct a cohort study in which we compare the social exclusion of older adults who were childless during a time when having children was the norm with those who had children during that same period (mechanism 1), and compare the social exclusion of older adults who were childless during a time when the norm was to have children with those who were childless during a period when the norm was less strict (mechanism 2). In a cohort comparison, comparing mutually exclusive groups of people exposed to different conditions is essential while keeping other relevant factors constant.

For the earlier-born cohort (Cohort 1), we use data from people between 65 and 74 participating in Round 1 (Edition 6.6; 2002), as these were between 20 and 28 years old during the post-World War II baby boom in Europe (Sánchez-Barricarte 2018). For the later-born cohort, we use data from people of the same age participating in Round 9 (Edition 3.1; 2018), the latest suitable wave since the start, to maximize the historical time between the cohorts. We did not use the latest data collection (Round 10), as that was during the pandemic when many people were socially isolated due to the social distancing regulations. The age range of eight years corresponds with the years most people transition into parenthood. While people can transition into parenthood beyond these limits, expanding the years would result in an overlap between the cohorts or a shorter historical time difference between them. The minimum age of 65 for the respondents in the two birth cohorts was based on the empirical finding that the network size often changes remarkably during retirement age (Suanet and Huxhold 2020), and 65 is the age at which people have retired in most countries.

We conducted separate analyses for the two dependent variables: (1) meeting with friends, relatives, or colleagues and (2) participating in social activities. In line with other scholars (e.g. Jackson 1999; Walsh et al. 2017), we decided not to dichotomize SE as that would suggest a sharp demarcation between in- and exclusion, which we believe is an unjust simplification. For each outcome variable, two models were constructed. The first model (Model 1) included the independent and control variables. The second model (Model 2) added three interaction terms to examine potential cohort and gender differences and differences according to partner status in the association between childlessness status and the respective outcome variables.

The data were analysed using linear regression models. Although the meetings and social activities scale is technically ordinal, it approximates a continuous measure given the number of categories and the nature of the data distribution (e.g. Norris et al. 2006; Lunt 2015). The assumption of interval-level measurement, while not strictly met, is considered reasonable in this case because the scale covers a wide range of behaviours with nearly equidistant points. In addition, we conducted a sensitivity analysis using ordinal logistic regression to ensure our results were consistent across different modelling approaches. The results were qualitatively similar, suggesting that the choice of method did not substantially alter our conclusions. To investigate gender-specific trends in the association between childlessness and SE and cohort and partner status differences in this association, we ran the same two interaction models as in the overall sample, but stratified the analysis by gender. All analyses were conducted in RStudio.

### Sensitivity analysis

To further investigate the stability of our results, we conducted a sensitivity analysis focusing exclusively on the variables of interest, namely childlessness, cohort, gender, and their interaction terms. As these variables are central to understanding the variations in the outcome across countries, we subjected them to varying assumptions and parameter values in the analysis. The sensitivity analysis yielded results that were consistent with the original model. Specifically, the direction of the associations remained the same. This affirms the reliability and robustness of our findings. Moreover, we ran a model including all countries of the ESS participating in both rounds (see Table 10 in Appendix A). The main effects remained the same as in the analytical sample, further affirming the robustness of our findings.

## Results

### Descriptive statistics

Compared to the people with children, those without had a lower frequency of contact with friends, relatives, or colleagues, took less often part in social activities, were slightly older, more often male, had lower education, and were more often without a partner. Compared to the earlier-born cohort, the later-born cohort met more often with friends, relatives, and colleagues, was more socially active, slightly younger, and highly educated, and had better subjective health, and the percentage of people with migrant backgrounds was higher. Compared to men, women were less educated, lived less often with partners, and had lower subjective health.

### Linear regression analysis

Table 2 presents the results of the linear regression models. Our first research question concerned whether childless older individuals are more prone to exclusion from meetings with friends, relatives, and colleagues and exclusion from social activities than those with children. For meetings, Model 1 showed an estimate for being childless of − 0.08 (CI: − 0.14, − 0.02, *p* < 0.01), indicating that those who were childless had fewer social meetings than parents. For social activities, Model 1 showed an estimate for being childless of − 0.10 (CI: − 0.16, − 0.04, *p* < 0.01), indicating that childless people had fewer social activities than parents. This confirmed our first hypothesis that childless older adults are more socially excluded than older parents.

**Table 2:** Linear regression analysis (weighted data)

	Meetings						Activities					
*Predictors*	*Estimates*	*CI*	*p*	*Estimates*	*CI*	*p*	*Estimates*	*CI*	*p*	*Estimates*	*CI*	*p*
(Intercept)	− 0.51	− 0.65 to − 0.38	** < 0.001**	− 0.50	− 0.63 to − 0.36	** < 0.001**	− 1.35	− 1.48 to − 1.21	** < 0.001**	− 1.32	− 1.46 to − 1.18	** < 0.001**
Childless	− 0.08	− 0.14 to − 0.02	**0.009**	− 0.13	− 0.25 to − 0.01	**0.039**	− 0.10	− 0.16 to − 0.04	**0.002**	− 0.20	− 0.33 to − 0.07	**0.002**
Female	0.05	0.01–0.10	**0.026**	0.03	− 0.02–0.09	0.255	0.10	0.05–0.14	** < 0.001**	0.08	0.02–0.13	**0.006**
Born in country	− 0.01	− 0.11––0.09	0.822	− 0.01	− 0.11–0.09	0.818	− 0.02	− 0.12–0.08	0.643	− 0.03	− 0.12–0.07	0.611
Education	0.04	0.02–0.06	** < 0.001**	0.04	0.02–0.06	** < 0.001**	0.09	0.08–0.11	** < 0.001**	0.09	0.08–0.11	** < 0.001**
Self-rated health	0.13	0.10–0.17	** < 0.001**	0.13	0.10–0.17	** < 0.001**	0.20	0.16–0.23	** < 0.001**	0.20	0.16–0.23	** < 0.001**
Later-born cohort	0.01	− 0.04–0.06	0.703	0.02	− 0.04–0.07	0.583	0.11	0.06–0.16	** < 0.001**	0.11	0.05–0.17	** < 0.001**
Has partner	− 0.13	− 0.18 to − 0.07	** < 0.001**	− 0.14	− 0.19–− 0.08	** < 0.001**	0.05	− 0.00–0.10	0.062	0.02	− 0.04–0.08	0.498
*Hampered in daily activities (HDA) (ref.: yes)*												
HDA to some extent	− 0.02	− 0.12–0.08	0.751	− 0.02	− 0.11–0.08	0.758	0.24	0.14–0.34	** < 0.001**	0.24	0.14–0.34	** < 0.001**
No HDA	0.01	− 0.09–0.11	0.866	0.01	− 0.09–0.11	0.857	0.30	0.19–0.40	** < 0.001**	0.30	0.19–0.40	** < 0.001**
*Country groups (ref.: conservative)*												
Social-democratic	0.17	0.11–0.23	** < 0.001**	0.17	0.11–0.23	** < 0.001**	0.07	0.01–0.13	**0.020**	0.07	0.01–0.13	**0.018**
Liberal	0.07	0.01–0.14	**0.035**	0.08	0.01–0.14	**0.027**	0.03	− 0.04–0.10	0.449	0.03	− 0.04–0.10	0.374
South European	0.06	− 0.01–0.14	0.103	0.06	− 0.01–0.14	0.100	− 0.03	− 0.11–0.04	0.411	− 0.03	− 0.11–0.05	0.419
*Interaction effects*												
Childless x later-born cohort				− 0.03	− 0.14–0.09	0.635				0.01	− 0.10–0.13	0.817
Childless x female				0.10	− 0.01–0.22	0.083				0.07	− 0.04–0.19	0.214
Childless x partner				0.04	− 0.08–0.16	0.524				0.11	− 0.01–0.23	0.065
Observations	6227			6227			6150			6150		
R^2^ /R^2^ adjusted	0.033/0.031			0.034/0.031			0.093/0.092			0.094/0.092		

*p*-values < 0.05 are shown in bold

Our second research question was whether the differences in SE between childless older people and older parents differed for later-born cohorts. This question was answered by adding an interaction term to the model (Model 2). The estimate for the interaction effect between childlessness and birth cohort was not statistically significant (CI: − 0.14, 0.09, *p* = 0.64) for meetings and not significant for social activities (CI: − 0.10, 0.13, *p* = 0.82), indicating that childless older people from earlier-born cohorts were not more socially excluded than childless older people in later-born cohorts. Our second hypothesis was rejected.

Our third question concerned a gender difference in the associations between childlessness and SE. We added a second interaction term to the model (Model 2), i.e. the interaction between gender and childlessness, tested simultaneously with the first interaction term. This interaction was insignificant for meetings (CI: − 0.01, 0.22, *p* = 0.08) and for social activities (CI: − 0.04, 0.19, *p* = 0.21). We rejected our third hypothesis.

The fourth hypothesis concerned a difference between childless older adults regarding their partner status. We added a third interaction term to the model (Model 2) and tested it simultaneously with the two other interaction terms. This interaction was insignificant for meetings (CI: − 0.08, 0.16, *p* = 0.52) and social activities (CI: − 0.01, 0.23, *p* = 0.06). We rejected our fourth hypothesis.

The results for the male subsample are presented in Table 3. With regard to our research question, if the association between childlessness and SE is different between the earlier- and later-born cohort according to gender, results for the male subsample revealed that in the model investigating meetings, there was no significant difference between childless men from the earlier- and those from the later-born cohort (CI: − 0.21, 0.11, *p* = 0.56). In the model investigating social activities, there was also no significant difference between childless men from the earlier- and those from the later-born cohort (CI: − 0.09, 0.24, *p* = 0.37). We, therefore, rejected our fifth hypothesis (5a). With regard to partner status, the interaction term was insignificant in both models. Childless men with a partner did not meet more than those without a partner (CI: − 0.10, 0.24, *p* = 0.41) and did not have more social activities than those without a partner (CI: − 0.10, 0.24, *p* = 0.40).

**Table 3:** Linear regression analysis male subsample (weighted data)

	Meetings						Activities					
*Predictors*	*Estimates*	*CI*	*p*	*Estimates*	*CI*	*p*	*Estimates*	*CI*	*p*	*Estimates*	*CI*	*p*
(Intercept)	− 0.46	− 0.65 to − 0.27	** < 0.001**	− 0.45	− 0.65 to − 0.25	** < 0.001**	− 1.30	− 1.49 to − 1.10	** < 0.001**	− 1.27	− 1.47 to − 1.07	** < 0.001**
Childless	− 0.13	− 0.21 to − 0.04	**0.003**	− 0.14	− 0.30–0.02	0.092	− 0.11	− 0.19 to − 0.02	**0.013**	− 0.20	− 0.36 to − 0.03	**0.021**
Born in Country	0.08	− 0.07–0.23	0.270	0.08	− 0.07–0.23	0.288	− 0.04	− 0.19–0.11	0.590	− 0.04	− 0.19–0.11	0.612
Education	0.02	− 0.00–0.05	0.094	0.02	− 0.00–0.05	0.096	0.06	0.04–0.09	** < 0.001**	0.06	0.04–0.09	** < 0.001**
Self-rated health	0.11	0.06–0.16	** < 0.001**	0.11	0.06–0.16	** < 0.001**	0.20	0.16–0.25	** < 0.001**	0.20	0.16–0.25	** < 0.001**
Later-born cohort	0.05	− 0.02–0.12	0.167	0.06	− 0.02––0.14	0.143	0.13	0.05–0.20	**0.001**	0.11	0.03–0.19	**0.010**
Has partner	− 0.07	− 0.15–0.01	0.067	− 0.09	− 0.19 to − 0.00	**0.050**	0.17	0.09–0.25	** < 0.001**	0.15	0.05–0.24	**0.002**
*Hampered in daily activities (HDA) (ref.: yes)*												
HDA to some extent	0.01	− 0.13–0.16	0.865	0.01	− − 0.13–0.16	0.841	0.11	− 0.03–0.26	0.131	0.12	− 0.03–0.26	0.129
No HDA	0.03	− 0.12–0.18	0.687	0.03	− 0.12–0.19	0.662	0.21	0.06––0.37	**0.008**	0.21	0.06–0.37	**0.008**
*Country groups (ref.: conservative)*												
Social-democratic	0.13	0.05–0.22	**0.002**	0.13	0.05–0.22	**0.002**	0.05	− 0.04–0.14	0.255	0.05	− 0.04–0.14	0.247
Liberal	0.08	− 0.02–0.17	0.129	0.08	− 0.02–0.18	0.109	0.02	− 0.08–0.12	0.665	0.02	− 0.08–0.12	0.640
South European	0.14	0.03–0.25	**0.012**	0.14	0.03–0.26	**0.011**	0.01	− 0.11–0.12	0.918	0.01	− 0.11–0.12	0.905
*Interaction effects*												
Childless x later-born cohort				− 0.05	− 0.21–0.11	0.561				0.08	− 0.09–0.24	0.372
Childless x has partner				0.07	− 0.10–0.24	0.414				0.07	− 0.10–0.24	0.403
Observations	3109			3109			3073			3073		
R^2^ /R^2^ adjusted	0.022/0.018			0.022/0.018			0.085/0.082			0.085/0.082		

*p*-values < 0.05 are shown in bold

The results for the female subsample are presented in Table 4. With regard to our research question, if the association between childlessness and SE is different between the earlier- and later-born cohort according to gender, results for the female subsample revealed that in the model investigating meetings, there was no significant difference between childless women from the earlier- and those from the later-born cohort (CI: − 0.17, 0.17, *p* = 0.99). In the model investigating social activities, there was also no significant difference between childless women from the earlier- and those from the later-born cohort (CI: − 0.23, 0.12, *p* = 0.53). We, therefore, rejected our fifth hypothesis (5b). With regard to partner status, the interaction term was insignificant in both models. Childless women with a partner did not meet more than those without a partner (CI: − 0.17, 0.16, p = 0.94) and did not have more social activities than those without a partner (CI: − 0.06, 0.29, p = 0.19).

**Table 4:** Linear regression analysis female subsample (weighted data)

	Meetings						Activities					
*Predictors*	*Estimates*	*CI*	*p*	*Estimates*	*CI*	*p*	*Estimates*	*CI*	*p*	*Estimates*	*CI*	*p*
(Intercept)	− 0.51	− 0.68 to − 0.34	** < 0.001**	− 0.51	− 0.69 to − 0.34	** < 0.001**	− 1.30	− 1.48 to − 1.12	** < 0.001**	− 1.30	− 1.47 to − 1.12	** < 0.001**
Childless	− 0.03	− 0.11–0.05	0.502	− 0.03	− 0.17–0.12	0.731	− 0.08	− 0.16–0.01	0.071	− 0.10	− 0.25–0.05	0.192
Born in Country	− 0.09	− 0.22–0.03	0.155	− 0.09	− 0.22–0.04	0.157	− 0.03	− 0.16–0.10	0.672	− 0.03	− 0.16–0.10	0.623
Education	0.06	0.03–0.08	** < 0.001**	0.06	0.03–0.08	** < 0.001**	0.12	0.10–0.15	** < 0.001**	0.12	0.10–0.15	** < 0.001**
Self-rated health	0.16	0.11–0.20	** < 0.001**	0.16	0.11–0.20	** < 0.001**	0.18	0.13–0.23	** < 0.001**	0.18	0.13–0.23	** < 0.001**
Later-born cohort	− 0.03	− 0.10–0.04	0.376	− 0.03	− 0.11–0.04	0.426	0.11	0.04–0.18	**0.003**	0.12	0.04–0.20	**0.003**
Has partner	− 0.16	− 0.23 to − 0.10	** < 0.001**	− 0.16	− 0.23 to − 0.09	** < 0.001**	− 0.04	− 0.10–0.03	0.296	− 0.06	− 0.13–0.02	0.131
*Hampered in daily activities (HDA) (ref.: yes)*												
HDA to some extent	− 0.06	− 0.19–0.08	0.420	− 0.06	− 0.19–0.08	0.420	0.34	0.21–0.48	** < 0.001**	0.34	0.21–0.48	** < 0.001**
No HDA	− 0.03	− 0.17–0.12	0.725	− 0.03	− 0.17–0.12	0.726	0.37	0.22–0.51	** < 0.001**	0.37	0.22–0.51	** < 0.001**
*Country groups (ref.: conservative)*												
Social-democratic	0.20	0.12–0.29	** < 0.001**	0.20	0.12–0.29	** < 0.001**	0.09	0.00–0.17	**0.042**	0.09	0.00–0.17	**0.041**
Liberal	0.08	− 0.02–0.17	0.105	0.08	− 0.02–0.17	0.106	0.04	− 0.06–0.14	0.403	0.04	− 0.05–0.14	0.374
South European	0.00	− 0.10–0.10	0.997	0.00	− 0.10–0.10	0.997	− 0.07	− 0.17–0.04	0.220	− 0.06	− 0.17–0.04	0.231
*Interaction effects*												
Childless x later-born cohort				− 0.00	− 0.17–0.17	0.997				− 0.06	− 0.23–0.12	0.525
Childless x has partner				− 0.01	− 0.17–0.16	0.935				0.12	− 0.06–0.29	0.186
Observations	3118			3118			3077			3077		
R^2^ /R^2^ adjusted	0.053/0.049			0.053/0.049			0.112/0.109			0.112/0.109		

*p*-values < 0.05 are shown in bold

To account for heterogeneity between country groups, we also ran a set of stratified analyses by country group (results can be found in Appendix A). In conservative countries (Austria, Belgium, Switzerland, Germany, France, the Netherlands), the effect of childlessness was not significant for meetings (CI: − 0.17, 0.01, *p* = 0.072), but was significant for activities (estimate: − 0.10, CI: − 0.19, − 0.01, *p* = 0.025), indicating that childless individuals participated less in social activities than parents. In social-democratic countries (Denmark, Finland, Norway, Sweden), childlessness did not significantly impact meetings (CI: − 0.21, 0.02, *p* = 0.111), but had a significant negative effect on activities (estimate: − 0.16, CI: − 0.30, − 0.03, *p* = 0.017). In liberal countries (UK, Ireland), the effect of childlessness was not significant for meetings (CI: − 0.12, 0.16, *p* = 0.795), but for activities, childlessness had a significant negative effect (estimate: − 0.36, CI: − 0.65, − 0.08, *p* = 0.013). In southern European countries (Spain, Italy), the effect of childlessness was marginally significant for meetings (estimate: − 0.15, CI: − 0.31, 0.01, *p* = 0.066), showing a potential trend towards fewer meetings for childless individuals, but it was not significant for activities (CI: − 0.18, 0.10, *p* = 0.542).

The interaction effects between childlessness and key variables were also examined by country group. In conservative countries, none of the interaction terms was significant. Similarly, in social-democratic countries (Denmark, Finland, Norway, Sweden), no significant interaction effects were found. In liberal countries (UK, Ireland), however, there were significant interaction effects. The interaction between childlessness and gender was significant for both meetings (estimate: 0.32, CI: 0.05, 0.59, *p* = 0.018) and activities (estimate: 0.35, CI: 0.07, 0.63, *p* = 0.014), indicating that childless men had fewer meetings and activities than their female counterparts. Additionally, the interaction between childlessness and having a partner was significant for activities, with an estimate of 0.37 (CI: 0.09, 0.66, *p* = 0.009), suggesting that childless individuals without a partner were less engaged in activities. In southern European countries (Spain, Italy), none of the interaction effects was significant. Overall, interaction effects were mostly insignificant across country groups, except in liberal countries where gender and partnership status played a moderating role in the effect of childlessness on meetings and social activities.

## Discussion

This study questioned whether childlessness contributes to SE in later life. Based on the different opportunities to socially connect between childless people and parents, changes in the normative pressure to transition to parenthood, and gender differences in private and public responsibilities, we expected that older childless people have fewer contacts with friends, relatives, and colleagues and were less socially active compared to older parents. We further predicted that older childless people from earlier-born cohorts were more excluded from social relationships and social activities than childless older people born later and that older childless women are more excluded than older childless men. Finally, we hypothesized that childless older adults without a partner would have higher levels of SE than those with a partner.

Our first hypothesis that older people without children also have fewer contacts with friends, relatives, and colleagues and are less socially active than people with children was in accord with our findings. Even though friends and relatives might compensate for the absence of children (Schnettler and Wöhler 2016; Deindl and Brandt 2017), this does not seem valid for older people living in the Northern and Western parts of Europe. This aligns with Dykstra’s (2006) suggestion that childlessness does not necessarily allow for greater sociability. Children seem to be an essential resource for social integration, and having them protects against SE. While our study does not reveal the specific mechanism through which having children might protect against SE in later life, it may be through the wider formal and informal social bridges that children provide (Hadley 2018).

Our second hypothesis was rejected. We did not find that childless older people from earlier-born cohorts were more socially excluded than those in later-born cohorts. This suggests that childlessness exerts a robust impact on SE not only for the baby boom generation but also for a later-born cohort. In line with other findings (Groenou and Deeg 2010; Ang 2019), we observed a general increase in meetings and social activities, which must be similar for older parents and older childless people as the gap in social activities between older parents and older childless people remained stable over the years.

We can think of two methodological explanations for our finding that differences in SE between older childless people and older parents did not fade over time. The first is that changes in norms were too small, or the time interval between the cohorts was too short to exert a significant effect. There is not much systematic knowledge about how and how fast social norms change, but in one report on changing gender norms, it was concluded that norm change tends to be very slow (Harper et al. 2020). Another explanation may be that other differences between the two cohorts may have suppressed any effects. Although we controlled for several factors that could lead to biased results, there could still be a range of other factors that correlate highly with our dependent and independent variables. For example, physical and mental health could impact both subjective health (Pinquart 2001) and participation in social relations and society beyond the effect of subjective health.

Our third hypothesis was rejected as we did not observe gender differences in SE among childless individuals. This lack of gender difference cannot be explained by the commonly discussed differences in education, subjective health, or partner status between men and women as we controlled for these factors. There are several potential explanations we can invoke. One possibility is that not having children indeed acted as the "ultimate liberation" (Peterson 2015) from caring tasks, particularly for women, allowing them to participate in society at similar levels as men. Another perspective is that the stigma of childlessness, which could be responsible for SE, is not inherently different for men and women. Different developments in men and women may have equalized potential gender differences in SE among childless individuals. For instance, while childlessness might have a lesser impact on men's social life compared to women's, it may significantly affect men's health more than women's, mainly when family formation does not occur (O’Flaherty et al. 2016). Poor health, in turn, leads to higher levels of SE in later life (Sacker et al. 2017). To draw more conclusive insights, further research is necessary to explore the plausibility of these suggestions.

Our fourth hypothesis (H4), suggesting that childless people without a partner have higher levels of SE than those with a partner, was also rejected. There was no significant difference between childless people with or without a partner. It seems that having a partner can therefore not protect against the detrimental effects of the absence of children with regard to SE. These results were also consistent in the gender-stratified analysis.

Our fifth hypothesis, proposing that any disadvantage for childless people compared to parents will diminish for women (H5a) and increase for men (H5b), was rejected. This conclusion was drawn from the finding that associations between childlessness and SE remained consistent over time, and there were no gender-specific differences in the strength of the associations between childlessness and SE. Consequently, the substantive interpretation suggests that the disadvantage of not having children compared to those with children is similar over time, regardless of gender. However, it is also plausible to consider that the time interval between the two cohorts was too short and potential changes too subtle to detect. Studies with longer time intervals might be needed to detect the impact of changing norms and changing work–family trajectories more effectively.

### Limitations

The present study has several limitations that need to be acknowledged. Firstly, our operationalization of SE is limited in scope, as we focused solely on the dimension of social relationships. It is essential to recognize that SE is a multidimensional concept, and our study only addresses the aspect related to social relationships. Moreover, we lack empirical information about the changes in social norms over time, particularly regarding attitudes towards childlessness. As a result, we had to make assumptions about the evolution of these norms, which may introduce potential biases into our findings. Moreover, when conducting cohort comparisons, it is inherent that we are dealing with different groups of individuals. Although we made efforts to control for relevant factors, there may still be unobserved differences between cohorts that could impact the reliability of our conclusions.

Despite these shortcomings, our study benefited from a substantial sample size derived from diverse European countries. These countries share similar economic, social, and cultural backgrounds and have experienced similar demographic histories, including a baby boom. These factors contribute to the validity of our findings and allow for generalizations within this specific context. However, caution should be exercised when extrapolating the results to other populations or contexts outside this scope.

### Conclusions

To conclude, using SE as a conceptual framework to look at the consequences of childlessness in later life allowed us to advance the understanding of the parenthood transition and its potential effects on social connectedness across the life cycle and into old age, while at the same time indicating that norms are relevant to consider when studying SE. Moreover, while exclusion in older age is predominantly explained through contextual factors, our study captured the potential relevance of an individual-level factor, childlessness. As the negative impact of SE is robust, lingers in time, and has a similar bearing on the genders, not having children can be delineated as one of the drivers of SE in later life. Given the current upward trends in delayed childbearing and the falling birth rates in Western countries, interventions aiming to reduce SE should take the effect of childlessness into account.

## Data Availability

Data from this study are available at https://www.europeansocialsurvey.org/.

## Appendix A

See Tables 5, 6, 7, 8, 9 and 10.

**Table 5:** Descriptive statistics of the full study sample for the sensitivity test, by cohort, gender, and childlessness (weighted data)

	Had children	Childless	Sig. Of Δ	Cohort 1, born between 1929 and 1937	Cohort 2, born between 1945 and 1953	Sig. Of Δ	Men	Women	Sig. Of Δ
Ever had children living in the household (% yes)				80.3	79.0		78.1	80.9	**
Frequency of socially meeting with friends, relatives or colleagues (Mean, 1–7)	4.68	4.63		4.60	4.73	**	4.69	4.65	
Take part in social activities compared to others of same age (Mean, 1–5)	2.70	2.60	**	2.55	2.78	**	2.70	2.67	
Age of respondent (Mean, years)	69.22	69.42	**	69.30	69.23		69.32	69.20	*
Gender (% female)	54.1	49.8	**	53.5	53.0				
Highest level of education ISCED (Mean, 1–5)	2.57	2.44	**	2.15	2.84	**	2.74	2.37	**
Has a partner (% yes)	71.6	53.7	**	65.7	69.5	**	76.6	60.4	**
Subjective health (Mean, 1–5)	3.40	3.38		3.31	3.46	**	3.46	3.33	**
Hampered in daily activities (Mean, 1–3)	2.52	2.54		2.50	2.55	**	2.55	2.51	**
Migrant background (% yes)	6.2	7.1		5.30	7.20	**	6.30	6.50	
N	6859	1763		3736	4885		4034	4586	

Sig. Of Δ = Significance of difference (based on T-test for Means, Chi-squared test for percentages), * = *p* < 0.05, ***p* < 0.01. Included countries: Austria, Belgium, Switzerland, Czechia, Germany, Denmark, Spain, Finland, France, UK, Hungary, Ireland, Italy, the Netherlands, Norway, Poland, Portugal, Sweden, and Slovakia

**Table 6:** Linear regression conservative countries (Austria, Belgium, Switzerland, Germany, France, and the Netherlands; weighted data)

	Meetings			Meetings			Activities			Activities		
*Predictors*	*Estimates*	*CI*	*p*	*Estimates*	*CI*	*p*	*Estimates*	*CI*	*p*	*Estimates*	*CI*	*p*
(Intercept)	-0.67	-0.88 – -0.45	** < 0.001**	-0.64	-0.86 – -0.43	** < 0.001**	-1.40	-1.62 – -1.18	** < 0.001**	-1.38	-1.60 – -1.15	** < 0.001**
Childless	-0.08	-0.17 – 0.01	0.072	-0.16	-0.35 – 0.03	0.100	-0.10	-0.19 – -0.01	**0.025**	-0.17	-0.37 – 0.02	0.082
Female	0.07	-0.01 – 0.14	0.088	0.03	-0.05 – 0.12	0.428	0.12	0.04 – 0.20	**0.002**	0.11	0.02 – 0.20	**0.013**
Born in Country	0.00	-0.12 – 0.13	0.956	0.00	-0.13 – 0.13	0.976	-0.01	-0.14 – 0.12	0.906	-0.01	-0.14 – 0.12	0.874
Education	0.05	0.02 – 0.08	**0.001**	0.05	0.02 – 0.08	**0.002**	0.13	0.10 – 0.16	** < 0.001**	0.13	0.10 – 0.16	** < 0.001**
Self-rated health	0.17	0.12 – 0.23	** < 0.001**	0.17	0.12 – 0.23	** < 0.001**	0.18	0.13 – 0.24	** < 0.001**	0.18	0.13 – 0.24	** < 0.001**
Later-born cohort	0.12	0.05 – 0.20	**0.001**	0.14	0.05 – 0.22	**0.002**	0.14	0.06 – 0.21	** < 0.001**	0.13	0.05 – 0.22	**0.003**
Has partner	-0.14	-0.22 – -0.06	** < 0.001**	-0.16	-0.25 – -0.07	** < 0.001**	0.02	-0.06 – 0.10	0.563	-0.00	-0.09 – 0.09	0.999
Hampered in daily activities (HDA) (ref.: yes)												
HDA to some extent	0.05	-0.10 – 0.20	0.526	0.05	-0.10 – 0.20	0.511	0.17	0.02 – 0.33	**0.029**	0.18	0.02 – 0.33	**0.029**
No HDA	0.01	-0.15 – 0.18	0.857	0.02	-0.14 – 0.18	0.832	0.24	0.07 – 0.40	**0.005**	0.24	0.07 – 0.40	**0.006**
Interaction effects												
Childless x later-born cohort				-0.07	-0.24 – 0.11	0.448				0.01	-0.17 – 0.19	0.949
Childless x female				0.13	-0.05 – 0.31	0.147				0.04	-0.15 – 0.22	0.707
Childless x has partner				0.10	-0.08 – 0.28	0.285				0.09	-0.09 – 0.28	0.324
Observations	2660			2660			2632			2632		
R^2^ / R^2^ adjusted	0.037 / 0.034			0.039 / 0.034			0.078 / 0.075			0.078 / 0.074		

*p*-values < 0.05 are shown in bold

**Table 7:** Linear regression social-democratic countries (Denmark, Finland, Norway, and Sweden; weighted data)

	Meetings						Activities					
*Predictors*	*Estimates*	*CI*	*p*	*Estimates*	*CI*	*p*	*Estimates*	*CI*	*p*	*Estimates*	*CI*	*p*
(Intercept)	− 0.12	− 0.34–0.10	0.299	− 0.12	− 0.34–0.11	0.313	− 1.31	− 1.57 to − 1.06	** < 0.001**	− 1.31	− 1.57 to − 1.05	** < 0.001**
Childless	− 0.09	− 0.21–0.02	0.111	− 0.10	− 0.34–0.14	0.409	− 0.16	− 0.30 to − 0.03	**0.017**	− 0.17	− 0.45–0.11	0.240
Female	0.13	0.05–0.21	**0.001**	0.13	0.05–0.22	**0.002**	0.14	0.05–0.23	**0.004**	0.14	0.04–0.24	**0.007**
Born in Country	− 0.13	− 0.34–0.08	0.212	− 0.13	− 0.33–0.08	0.235	0.04	− 0.20–0.28	0.758	0.04	− 0.21–0.28	0.764
Education	0.02	− 0.01–0.05	0.130	0.02	− 0.01–0.05	0.153	0.08	0.04–0.11	** < 0.001**	0.08	0.04–0.11	** < 0.001**
Self-rated health	0.09	0.03–0.15	**0.002**	0.09	0.03–0.15	**0.002**	0.23	0.17–0.30	** < 0.001**	0.23	0.17–0.30	** < 0.001**
Later-born cohort	0.04	− 0.05–0.13	0.357	0.02	− 0.07–0.12	0.589	0.05	− 0.05–0.15	0.297	0.05	− 0.05–0.16	0.321
Has partner	− 0.12	− 0.21 to − 0.04	**0.005**	− 0.10	− 0.20 to − 0.01	**0.030**	0.06	− 0.04–0.16	0.229	0.06	− 0.05–0.17	0.302
*Hampered in daily activities (HDA) (ref.: yes)*												
HDA to some extent	0.01	− 0.17–0.18	0.946	0.00	− 0.17–0.18	0.965	0.24	0.04–0.44	**0.020**	0.24	0.04–0.44	**0.020**
No HDA	0.02	− 0.16–0.20	0.802	0.02	− 0.16–0.20	0.818	0.31	0.10–0.52	**0.004**	0.31	0.10–0.52	**0.004**
*Interaction effects*												
Childless x later-born cohort				0.12	− 0.11–0.35	0.300				− 0.01	− 0.28–0.26	0.965
Childless x female				− 0.02	− 0.25–0.21	0.872				− 0.01	− 0.28–0.26	0.951
Childless x has partner				− 0.11	− 0.34–0.12	0.360				0.02	− 0.25–0.29	0.869
Observations	1606			1606			1589			1589		
R^2^ /R^2^ adjusted	0.029/0.023			0.030/0.023			0.109/0.103			0.109/0.102		

*p*-values < 0.05 are shown in bold

**Table 8:** Linear regression liberal countries (UK, Ireland; weighted data)

	Meetings						Activities					
*Predictors*	*Estimates*	*CI*	*p*	*Estimates*	*CI*	*p*	*Estimates*	*CI*	*p*	*Estimates*	*CI*	*p*
(Intercept)	− 0.11	− 0.40–0.18	0.452	− 0.02	− 0.31–0.28	0.915	− 1.13	− 1.43 to − 0.83	** < 0.001**	− 0.99	− 1.30 to − 0.68	** < 0.001**
Childless	0.02	− 0.12–0.16	0.795	− 0.20	− 0.47–0.08	0.155	− 0.05	− 0.19–0.10	0.531	− 0.36	− 0.65 to − 0.08	**0.013**
Female	0.07	− 0.04–0.19	0.206	− 0.01	− 0.14–0.12	0.882	0.10	− 0.02–0.22	0.094	0.00	− 0.13–0.14	0.943
Born in Country	0.02	− 0.20–0.24	0.856	0.02	− 0.20–0.25	0.835	− 0.12	− 0.35–0.11	0.304	− 0.12	− 0.35–0.11	0.310
Education	0.06	0.02–0.10	**0.002**	0.06	0.02–0.09	**0.004**	0.07	0.03–0.11	** < 0.001**	0.07	0.03–0.10	**0.001**
Self-rated health	0.08	0.00–0.15	**0.047**	0.08	0.00–0.15	**0.049**	0.15	0.07–0.22	** < 0.001**	0.14	0.06–0.22	** < 0.001**
Later-born cohort	− 0.22	− 0.34 to − 0.10	** < 0.001**	− 0.22	− 0.35 to − 0.08	**0.002**	− 0.00	− 0.13–0.12	0.942	− 0.00	− 0.14–0.14	0.966
Has partner	− 0.14	− 0.26 to − 0.02	**0.024**	− 0.19	− 0.33 to − 0.05	**0.007**	0.06	− 0.07–0.19	0.344	− 0.04	− 0.19–0.10	0.551
*Hampered in daily activities (HDA) (ref.: yes)*												
HDA to some extent	− 0.03	− 0.25–0.20	0.821	− 0.02	− 0.24–0.20	0.874	0.39	0.16–0.62	**0.001**	0.40	0.17–0.63	**0.001**
No HDA	0.05	− 0.18–0.28	0.667	0.05	− 0.18–0.27	0.686	0.50	0.27–0.74	** < 0.001**	0.50	0.27–0.74	** < 0.001**
*Interaction effects*												
Childless x later-born cohort				0.01	− 0.26–0.28	0.950				0.00	− 0.28–0.29	0.979
Childless x female				0.32	0.05–0.59	**0.018**				0.35	0.07–0.63	**0.014**
Childless x has partner				0.17	− 0.10–0.44	0.222				0.37	0.09–0.66	**0.009**
Observations	1067			1067			1062			1062		
R^2^ /R^2^ adjusted	0.031/0.022			0.037/0.026			0.090/0.082			0.100/0.090		

*p*-values < 0.05 are shown in bold

**Table 9:** Linear regression south European countries (Spain, Italy; weighted data)

	Meetings						Activities					
*Predictors*	*Estimates*	*CI*	*p*	*Estimates*	*CI*	*p*	*Estimates*	*CI*	*p*	*Estimates*	*CI*	*p*
(Intercept)	− 0.28	− 0.67–0.11	0.156	− 0.30	− 0.71–0.11	0.148	− 1.40	− 1.74 to − 1.06	** < 0.001**	− 1.41	− 1.77 to − 1.06	** < 0.001**
Childless	− 0.15	− 0.31–0.01	0.066	− 0.08	− 0.45–0.29	0.668	− 0.04	− 0.18–0.10	0.542	− 0.02	− 0.33–0.30	0.922
Female	− 0.10	− 0.25–0.04	0.159	− 0.12	− 0.29–0.05	0.160	− 0.02	− 0.15–0.10	0.706	− 0.02	− 0.17–0.12	0.742
Born in Country	− 0.08	− 0.64–0.48	0.774	− 0.09	− 0.65–0.48	0.764	0.00	− 0.47–0.48	0.985	0.00	− 0.47–0.48	0.987
Education	0.04	− 0.01–0.10	0.131	0.04	− 0.02–0.10	0.148	0.10	0.05–0.15	** < 0.001**	0.10	0.05–0.15	** < 0.001**
Self-rated health	0.18	0.07–0.28	**0.001**	0.18	0.07–0.28	**0.001**	0.23	0.14–0.31	** < 0.001**	0.23	0.14–0.32	** < 0.001**
Later-born cohort	− 0.09	− 0.23–0.06	0.248	− 0.05	− 0.22–0.11	0.533	0.30	0.18–0.43	** < 0.001**	0.31	0.17–0.45	** < 0.001**
Has partner	− 0.05	− 0.20–0.10	0.519	− 0.04	− 0.22–0.15	0.697	0.10	− 0.03–0.23	0.136	0.11	− 0.05–0.27	0.181
*Hampered in daily activities (HDA) (ref.: yes)*												
HDA to some extent	− 0.26	− 0.59–0.08	0.130	− 0.26	− 0.59–0.08	0.133	0.21	− 0.08–0.50	0.160	0.21	− 0.08–0.51	0.159
No HDA	− 0.09	− 0.43–0.25	0.600	− 0.09	− 0.43–0.25	0.619	0.12	− 0.18–0.41	0.439	0.12	− 0.18–0.42	0.436
*Interaction effects*												
Childless x later-born cohort				− 0.13	− 0.46–0.19	0.423				− 0.02	− 0.30–0.26	0.870
Childless x female				0.08	− 0.24–0.41	0.620				0.01	− 0.27–0.28	0.961
Childless x has partner				− 0.05	− 0.39–0.28	0.755				− 0.03	− 0.31–0.26	0.853
Observations	894			894			867			867		
R^2^ /R^2^ adjusted	0.038/0.028			0.039/0.026			0.117/0.107			0.117/0.104		

*p*-values < 0.05 are shown in bold

**Table 10:** Linear regression including all countries participating in Rounds 1 and 9 of the ESS (weighted data)

	Meetings						Activities					
*Predictors*	*Estimates*	*CI*	*p*	*Estimates*	*CI*	*p*	*Estimates*	*CI*	*p*	*Estimates*	*CI*	*p*
(Intercept)	-0.47	-0.59 – -0.36	** < 0.001**	-0.44	-0.56 – -0.33	** < 0.001**	-1.27	-1.38 – -1.15	** < 0.001**	-1.24	-1.36 – -1.11	** < 0.001**
Childless	-0.06	-0.11 – -0.01	**0.021**	-0.16	-0.27 – -0.05	**0.004**	-0.10	-0.15 – -0.04	** < 0.001**	-0.20	-0.32 – -0.09	** < 0.001**
Female	0.05	0.01 – 0.09	**0.016**	0.04	-0.01 – 0.08	0.120	0.07	0.03 – 0.12	**0.001**	0.06	0.01 – 0.11	**0.016**
Born in country	0.04	-0.04 – 0.13	0.342	0.04	-0.05 – 0.13	0.350	-0.02	-0.10 – 0.07	0.737	-0.02	-0.10 – 0.07	0.731
Education	0.04	0.02 – 0.05	** < 0.001**	0.04	0.02 – 0.05	** < 0.001**	0.10	0.08 – 0.12	** < 0.001**	0.10	0.08 – 0.12	** < 0.001**
Self-rated health	0.15	0.12 – 0.18	** < 0.001**	0.15	0.12 – 0.18	** < 0.001**	0.20	0.17 – 0.23	** < 0.001**	0.20	0.17 – 0.23	** < 0.001**
Later-born cohort	0.00	-0.04 – 0.05	0.869	-0.00	-0.05 – 0.04	0.849	0.08	0.04 – 0.12	** < 0.001**	0.07	0.02 – 0.12	**0.006**
Has partner	-0.11	-0.16 – -0.07	** < 0.001**	-0.14	-0.19 – -0.09	** < 0.001**	0.06	0.02 – 0.10	**0.009**	0.04	-0.01 – 0.09	0.162
Hampered in daily activities (HDA) (ref.: yes)												
HDA to some extent	-0.00	-0.08 – 0.08	0.965	-0.00	-0.08 – 0.08	0.944	0.19	0.11 – 0.28	** < 0.001**	0.19	0.11 – 0.27	** < 0.001**
No HDA	0.00	-0.08 – 0.09	0.940	0.00	-0.08 – 0.09	0.963	0.26	0.17 – 0.35	** < 0.001**	0.26	0.17 – 0.35	** < 0.001**
Country groups (ref.: conservative)												
Social-democratic	0.16	0.10 – 0.22	** < 0.001**	0.16	0.11 – 0.22	** < 0.001**	0.07	0.01 – 0.13	**0.022**	0.07	0.01 – 0.13	**0.022**
Liberal	0.07	-0.00 – 0.13	0.051	0.07	0.00 – 0.14	**0.041**	0.03	-0.04 – 0.10	0.427	0.03	-0.04 – 0.10	0.384
South European	0.15	0.08 – 0.22	** < 0.001**	0.15	0.08 – 0.21	** < 0.001**	-0.01	-0.08 – 0.06	0.715	-0.02	-0.09 – 0.05	0.642
East European	-0.57	-0.63 – -0.51	** < 0.001**	-0.57	-0.63 – -0.51	** < 0.001**	-0.19	-0.25 – -0.13	** < 0.001**	-0.19	-0.25 – -0.13	** < 0.001**
Interaction effects												
Childless x later-born cohort				0.04	-0.07 – 0.14	0.501				0.05	-0.05 – 0.16	0.321
Childless x female				0.05	-0.05 – 0.15	0.309				0.06	-0.05 – 0.16	0.290
Childless x has partner				0.10	-0.00 – 0.20	0.051				0.09	-0.01 – 0.20	0.083
Observations	8213			8213			8079			8079		
R^2^ / R^2^ adjusted	0.109 / 0.107			0.109 / 0.107			0.108 / 0.107			0.109 / 0.107		

*p*-values < 0.05 are shown in bold

Countries include Austria, Belgium, Switzerland, Czechia, Germany, Denmark, Spain, Finland, France, UK, Hungary, Ireland, Italy, the Netherlands, Norway, Poland, Portugal, Sweden, and Slovakia
